# Genome-Wide Association Study for Seed Yield of Tepary Bean Using Whole-Genome Resequencing

**DOI:** 10.3390/ijms252011302

**Published:** 2024-10-21

**Authors:** Waltram Ravelombola, Aurora Manley, Hanh Pham, Madeline Brown, Caroline Ruhl, Protik Ghosh

**Affiliations:** 1 Texas A&M AgriLife Research, 11708 Highway 70 South, Vernon, TX 76384, USA; 2 Soil and Crop Sciences, Texas A&M University, 370 Olsen Blvd., College Station, TX 77843, USA; 3 Texas A&M AgriLife Research, 1102 East Drew Street, Lubbock, TX 79403, USA

**Keywords:** tepary bean, *Phaseolus acutifolius*, seed, yield, genomics

## Abstract

Tepary bean (*Phaseolus acutifolius* A. Gray) is a diploid legume species (2*n* = 2*x* = 22). It is the most drought- and heat-tolerant crop of the genus *Phaseolus*. Tepary bean is native to the northern part of Mexico and the south-western part of the U.S. The lack of molecular markers associated with agronomic traits such as 100-seed weight and seed yield limit the development of elite tepary bean cultivars. Therefore, the objectives of this study were to evaluate tepary bean for 100-seed weight and yield, and identify single-nucleotide polymorphism (SNP) markers associated with these traits. A total of 230,000 high-quality SNPs obtained from the whole-genome resequencing of 153 tepary bean accessions were used for this study. For 100-seed weight, a total of 5 and 20 SNPs were found using a mixed linear model (MLM) and compressed mixed linear model (cMLM), respectively. A candidate gene, *Phacu.CVR.002G320800.13*, encoding the squamosa promoter-binding protein-like (SBP domain) transcription factor family protein was found to be associated with 100-seed weight. For seed yield, a total of one and eight SNPs were identified using an MLM and cMLM, respectively. *Phacu.CVR.009G294200.1*, encoding for peroxidase family protein, was identified as a candidate gene for seed yield. Both *Phacu.CVR.002G320800.13* and *Phacu.CVR.009G294200.1* are likely to be involved in seed development of tepary bean. This is one of the few studies investigating the genetics of 100-seed weight and seed yield in tepary bean.

## 1. Introduction

Tepary bean (*Phaseolus acutifolius* A. Gray) is a diploid legume species (2*n =* 2*x =* 22) and belongs to the genus *Phaseolus* [1]. Its genome size is approximately 647 Mbp [2]. The name tepary is from the Spanish word ‘tepari’, which has been used to differentiate this crop from common bean (*Phaseolus vulgaris* L.) [3]. Tepary bean seeds are smaller than those of common bean or lima bean (*Phaseolus lunatus* L.) [4]. Mature petioles of tepary bean are also significantly smaller than those of common bean or lima bean [4]. Tepary bean also has a truncated first pair of aerial leaves, while the ones for common bean or lima bean are cordate [4]. Overall, tepary bean flowers are smaller than those of common bean or lima bean [5]. The upper part of tepary bean petals is united; however, it is separate for common bean [5].

Tepary bean is native to the northern part of Mexico and the south-western part of the U.S. [6,7,8]. A study reported that tepary bean was first planted by the Papago tribe of the Baboquivari and Quijotoa districts in the southern U.S. [4]. Tepary bean can associate with soil bradyrhizobial bacteria to establish root nodules and fix atmospheric nitrogen, which can increase soil fertility and crop productivity [9]. Tepary bean is also a nutrient-dense crop and a health-promoting food. It provides protein and other essential elements for human consumption and is used as forage for livestock [5,10,11]. In addition, tepary bean can be used as a summer crop and in rotation with winter crop production. The integration of summer legumes into the wheat (*Triticum aestivum* L.) cropping system has been shown to increase soil fertility [12]. Tepary bean is an excellent donor of various traits, including drought and heat tolerance, to common bean. Tepary bean is the most heat-resistant species of the genus *Phaseolus* [13]. Previous studies reported drought-tolerant interspecific lines obtained from introgression of the tepary bean drought tolerance trait to common bean [14,15].

Seed yield and 100-seed weight are important agronomic traits of tepary bean [2]. Seed yield directly reflects the crop production potential and has been a major trait of interest for crop improvement. Hundred-seed weight is another important trait because it is a yield component trait. However, strategies aiming to improve these traits remain limited in tepary bean. In addition, genetic information for these traits is still limited in tepary bean, slowing crop improvement, genetic diversity establishment, and breeding. In addition, studies investigating molecular markers associated with 100-seed weigh and seed yield are limited. Single-nucleotide polymorphisms (SNPs) are currently one of the most widely used molecular markers [13]. Single-nucleotide polymorphism (SNP) is a single variation in nucleotides between DNA sequences among organisms [16]. SNP markers have been utilized in various genetic studies to advance our understanding of the genetics controlling traits because they are high-throughput and cost-effective [17]. In the past decade, SNPs have been successfully used to conduct genetic mapping and candidate gene discovery [18]. SNPs can be identified using whole-genome resequencing. This approach allows for the discovery of a large set of SNPs, increasing the mapping resolution [19]. One of the mapping approaches using SNPs is genome-wide association studies (GWASs). Multiple GWASs have been successfully using SNPs derived from whole-genome resequencing of various plants and crops such as *Arabidopsis* [20], barley (*Hordeum vulgare* L.) [21], canola (*Brassica napus* L.) (Zhang et al., 2015) [22], common bean [23], cowpea (*Vigna unguiculata* L. Walp) [12], rice (*Oryza sativa* L.) [24], soybean (*Glycine max* L. Merr.) [25], etc. A genome-wide association study for resistance to weevils, common bacterial blight, Fusarium wilt, and bean common mosaic necrosis virus was conducted for tepary bean using a genotyping-by-sequencing approach [26]. However, a GWAS approach using whole-genome resequencing is still significantly lacking for tepary bean. Whole-genome resequencing can be used to develop SNP markers for agronomic traits in tepary bean. Therefore, the objectives of this study were to conduct a genome-wide association study (GWAS) for 100-seed weight and seed yield and to identify SNP markers associated with these traits in tepary bean.

## 2. Results

### 2.1. Phenotypic Data, Genetic Diversity, and Population Structure

**Figure 1:**
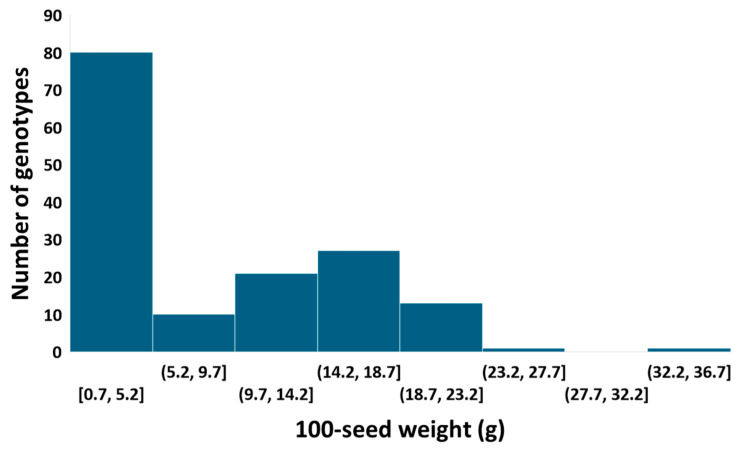
Variation in 100-seed weight among tepary bean accessions. The x-axis represents the variation in 100-seed weight among the tepary bean genotypes, and the y-axis represents the number of genotypes.

A large variation in 100-seed weight was identified among the tepary bean accessions evaluated in this study. The distribution of 100-seed weight was skewed to a lower seed weight (Figure 1), with a skewness coefficient of 0.67. For the combined 2021 and 2022 data, the 100-seed weight varied from 0.7 g to 32.2 g, with an average of 8.5 g and a standard deviation of 6.8 g. Hundred-seed weight was significantly different among the tepary bean accessions (*p*-value < 0.0001). The accessions with the highest 100-seed weight were PI331405 (32.2 g), PI196932 (26.8 g), PI535231 (22.7 g), PI440795 (22.4 g), W632483 (20.8 g), PI440801 (19.9 g), PI502217 (19.9 g), PI535227 (19.9 g), PI661512 (19.9 g), and PI440786 (19.3 g). These accessions can be a good source for large seeds traits for tepary bean genetic improvement. The accessions with the smallest 100-seed weight were PI640955 (2.1 g), PI640956 (2.1 g), PI640992 (2.1 g), PI535253 (2.1g), PI535255 (2.1 g), PI535254 (2.0 g), PI535373 (1.8 g), PI535239 (1.8 g), PI653236 (1.8 g), and PI691871 (0.7 g).

**Figure 2:**
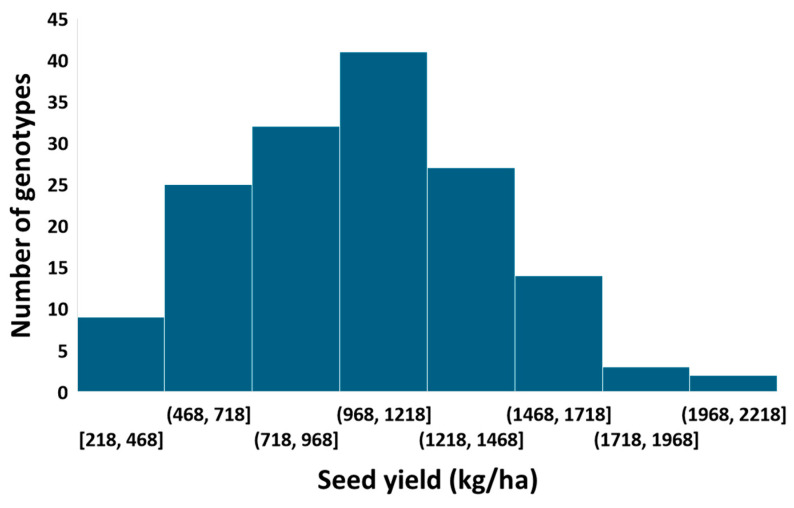
Variation in seed yield among tepary bean accessions. The x-axis represents the variation in seed yield among the tepary bean genotypes, and the y-axis represents the number of genotypes.

A large variation in seed yield was also found among the tepary accessions. The distribution of seed yield was normal (Figure 2), with a skewness coefficient of 0.08. For the combined 2021 and 2022 data, the seed yield varied from 217 kg/ha to 2147 kg/ha, with an average of 1031 kg/ha and a standard deviation of 380 kg/ha. The seed yield was significantly different among the tepary bean accessions (*p*-value < 0.0001). The accessions with the highest seed yield were W638695 (2147 kg/ha), PI638928 (2088 kg/ha), PI406633 (1908 kg/ha), PI638838 (1826 kg/ha), PI653249 (1728 kg/ha), PI440806 (1717 kg/ha), PI638835 (1704 kg/ha), W638766 (1694 kg/ha), PI638912 (1673 kg/ha), and PI642116 (1630 kg/ha). These accessions can be a good parent to improve the seed yield of tepary bean. The accessions with the lowest seed yield were PI638927 (478 kg/ha), PI535261 (445 kg/ha), PI197032 (425 kg/ha), PI640986 (416 kg/ha), PI666350 (402 kg/ha), W632480 (399 kg/ha), W626190 (333 kg/ha), PI535262 (298 kg/ha), PI640987 (290 kg/ha), and PI653236 (218 kg/ha).

**Figure 3:**
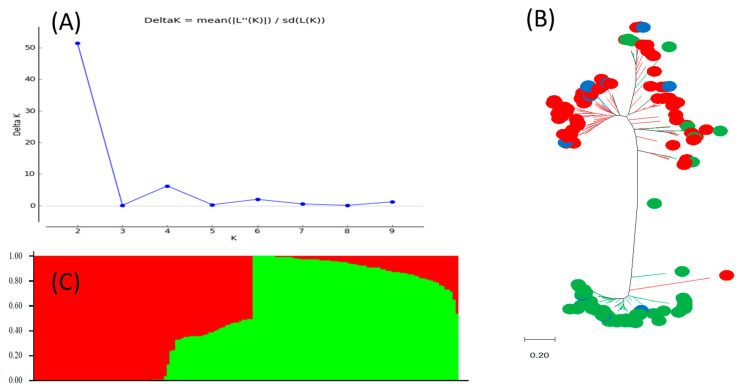
Genetic diversity and population structure at K = 2. (**A**) Delta K values were determined by Structure Harvester for various numbers of populations assumed (K) in STRUCTURE analysis. (**B**) Population structure of 153 tepary bean accessions divided into two subpopulations using STRUCTURE 2.3.4, where each accession is shown on the x-axis and the subgroup membership is displayed on the y-axis. The color coding (Q1: red; Q2: green) indicates the dispersion of the accessions into distinct subpopulations. (**C**) Maximum likelihood (ML) phylogenetic tree of the 153 tepary bean accessions drawn using MEGA 7. The color codes are consistent with those in (**A**,**B**). Admixture is shown in blue.

Figure 3 shows the genetic diversity and population structure among the 153 tepary bean accessions. The population structure analysis showed that these tepary bean accessions contain two sub-populations, Q1 and Q2, with a low level of admixture. Q1 consists of 71 tepary bean accessions (46%). The origins of the Q1 accessions are Argentina (1), El Salvador (7), Ethiopia (1), Mexico (20), Morocco (1), Nicaragua (2), and the U.S. (28). The origin of the 10 accessions from the Q1 group is unknown. The Q2 subpopulation has 72 accessions (47%). The Q2 accessions mainly originated from Mexico (26) and the U.S. (23), with 5 accessions with unknown origins for that group. The admixture group Q1Q2 only has 10 accessions (7%) that originated from Mexico (2) and the U.S. (8). The results indicate that the Q1 group has richer geographical diversity compared the Q2 subpopulation and the admixture Q1Q2, which only have accessions from Mexico and the U.S. A good correlation was also found between the population structure and the phylogenetic diversity tree. The genetic diversity results clearly grouped the two subpopulations into two distinct groups. However, a few misclassifications were found. Six accessions from the Q2 group were displayed among the Q1 cluster. Only one accession from the Q1 group was classified among the Q2 cluster. The accessions belonging to the admixture were scattered across the genetic diversity tree. Eight accessions from the admixture were shown among the Q1 cluster, and two accessions were displayed in the Q2 cluster. The results showed a good consistency between population structure and genetic diversity.

### 2.2. GWAS for 100-Seed Weight

**Figure 4:**
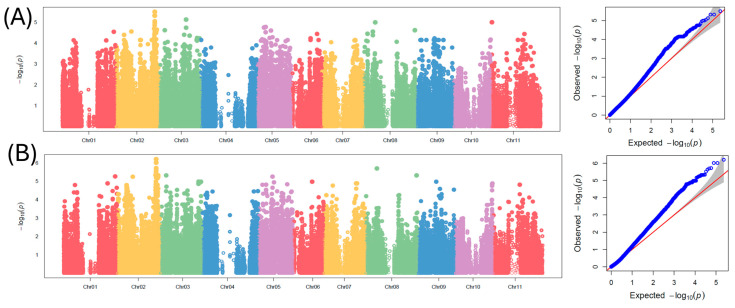
Manhattan and QQ plots for genome-wide association study for 100-seed weight in tepary bean: (**A**) mixed linear model (MLM) and (**B**) compressed mixed linear model (cMLM). For the Manhattan plot, chromosomes are represented on the x-axis, and the y-axis refers to the −log_10_(*p*) value of each SNP.

Table 1 and Figure 4 show the SNP markers that were found to be significantly associated with 100-seed weight in tepary bean using a mixed linear model (MLM) and compressed mixed linear model (cMLM). The MLM identified a total of five significant SNPs with large effects (>12%) for 100-seed weight. These SNPs consisted of Ph_Chr02_41007928 (LOD = 5.04, R-square = 12.72%), Ph_Chr02_41008221 (LOD = 5.32, R-square = 13.66%), Ph_Chr02_41008236 (LOD = 5.32, R-square = 13.66%), Ph_Chr02_41016239 (LOD = 5.50, R-square = 14.16%), and Ph_Chr03_29040578 (LOD = 5.13, R-square = 17.69%). The cMLM identified a total of 20 SNPs associated with 100-seed weight in tepary bean. Three SNPs, Ph_Chr01_54553950 (LOD = 5.26, R-square = 16.66%), Ph_Chr03_6452621 (LOD = 5.32, R-square = 15.88%), and Ph_Chr05_15111527 (LOD = 5.25, R-square = 14.78%) were found on chromosomes 1, 3, and 5, respectively. Most of the SNPs associated with 100-seed weight were found on chromosome 2. One significant SNP, Ph_Chr02_16248843 (LOD = 5.25, R-square = 15.28%), was located at 16,248,843 bp of chromosome 2, and a 3 Mbp region of chromosome 2 harbored 14 SNPs consisting of Ph_Chr02_40340436 (LOD = 5.33, R-square = 15.28), Ph_Chr02_40901629 (LOD = 5.52, R-square = 11.69), Ph_Chr02_41007928 (LOD = 5.72, R-square = 12.97), Ph_Chr02_41008221 (LOD = 6.01, R-square = 13.28), Ph_Chr02_41008236 (LOD = 6.01, R-square = 14.22), Ph_Chr02_41008249 (LOD = 5.20, R-square = 12.25), Ph_Chr02_41008429 (LOD = 5.63, R-square = 13.22), Ph_Chr02_41016239 (LOD = 6.20, R-square = 14.66), Ph_Chr02_42360227 (LOD = 5.19, R-square = 11.78), Ph_Chr02_42377727 (LOD = 5.16, R-square = 11.88), Ph_Chr02_42378284 (LOD = 5.16, R-square = 11.88), Ph_Chr02_43137572 (LOD = 5.23, R-square = 10.66), and Ph_Chr02_43159031 (LOD = 5.33, R-square = 12.13). Three other significant SNPs, Ph_Chr08_11898325 (LOD = 5.70, R-square = 17.19), Ph_Chr08_53883571 (LOD = 5.32, R-square = 15.88), and Ph_Chr08_53883585 (LOD = 5.32, R-square = 15.88), were found on chromosome 8 of tepary bean. Candidate gene analysis was conducted using the SNP Ph_Chr02_41008236 that was found to be consistent across the two models. This SNP was located on exome 2 of the candidate gene *Phacu.CVR.002G320800.13*, which encodes the squamosa promoter-binding protein-like (SBP domain) transcription factor family protein.

### 2.3. GWAS for Seed Yield

**Figure 5:**
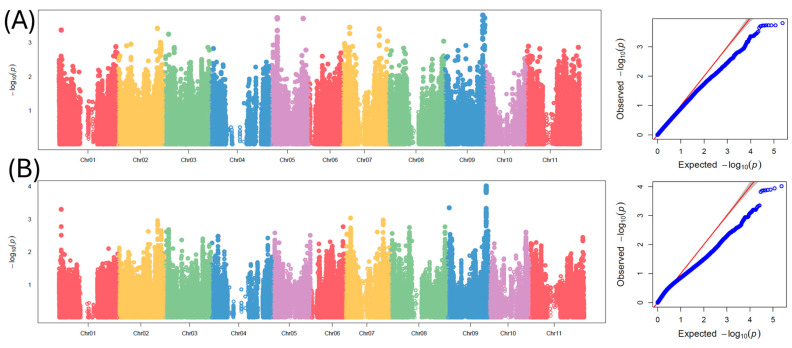
Manhattan and QQ plots for genome-wide association study for seed yield in tepary bean: (**A**) mixed linear model (MLM) and (**B**) compressed mixed linear model (cMLM). For the Manhattan plot, chromosomes are represented on the x-axis, and the y-axis refers to the −log_10_(p) value of each SNP.

Table 2 and Figure 5 show the SNP markers that were found to be significantly associated with seed yield in tepary bean using a mixed linear model (MLM) and compressed mixed linear model (cMLM). The MLM identified one SNP marker associated with seed yield in tepary. This SNP, Ph_Chr09_37443441 (LOD = 3.81), was located on chromosome 9 of tepary bean and contributed to 7.33% of the variation in seed yield. The cMLM identified a total of eight SNPs located on a 125 Kb region of chromosome 9. The SNPs consisted of Ph_Chr09_37317875 (LOD = 3.87, R-square = 6.10%), Ph_Chr09_37317968 (LOD = 3.87, R-square = 6.10%), Ph_Chr09_37321813 (LOD = 3.94, R-square = 6.10%), Ph_Chr09_37324571 (LOD = 3.86, R-square = 6.15%), Ph_Chr09_37332586 (LOD = 3.89, R-square = 6.08%), Ph_Chr09_37332627 (LOD = 3.82, R-square = 6.02%), Ph_Chr09_37443441 (LOD = 3.90, R-square = 6.12%), and Ph_Chr09_37443547 (LOD = 4.01, R-square = 6.21%). A candidate gene search was performed using the SNP Ph_Chr09_37443441 that was consistent across the two models. The candidate gene *Phacu.CVR.009G294200.1* was found 20 kb upstream of Ph_Chr09_37443441. This annotated gene encodes for a peroxidase superfamily protein.

## 3. Discussion

This study identified a large variation in the 100-seed weight and seed yield of tepary bean using two-year data. The results showed potential parents with larger seeds and a high yield that can be used to improve the agronomics of tepary bean. These lines can also be used as parents to build interspecific lines between tepary bean and common bean. We identified two subpopulations within the tepary bean panel being investigated. One of the subpopulations consisted of accessions from the U.S. and Mexico where tepary bean is native to [6,7,8]. The population structure information was used in the genome-wide association study analysis to control for false positive SNP discovery [12]. Genetic diversity can affect trait variation in this panel of tepary bean. However, the population structure and genetic diversity analysis were used to control for false positives that were attributed to population stratification rather than the genetic makeup of the population. The SNP effects of interest in this study were those that are not attributed to population stratification.

A genome-wide association study identified a total of 20 significant SNP markers associated with 100-seed weight in tepary bean. These SNPs had higher R-square values (>12%), indicating that tepary bean 100-seed weight can be controlled by fewer genes with major effects. Similar findings were also reported in other crops such as soybean [27]. The SNP Ph_Chr02_41008236 is located on the second exome of the annotated gene *Phacu.CVR.002G320800.13* that encodes for the squamosa promoter-binding protein-like (SBP domain) transcription factor family protein. This protein has been demonstrated to regulate plant growth and development [28]. This gene has been shown to be involved in 1000-kernel weight and kernel weigh formation in wheat [28]. This protein binds to genes involved in the ethylene pathways regulating spike development in wheat, and enhanced their expression [28]. This protein has been also shown to regulate seed development and size in *Arabidopsis* [29]. These findings suggest that *Phacu.CVR.002G320800.13* could be involved in regulating seed size in tepary bean. The identified SNP markers can be used as molecular tools to screen for larger seeds by targeting alleles conferring higher 100-seed weights. This will reduce the need for phenotyping of 100-seed weight and can help with efficiently and rapidly advancing population and generations when selection for 100-seed weight. In addition, the SNPs found within the candidate gene structure can be further used in functional genomic studies. They provide significant biological information such as the potential change in amino acid sequence due to the change in the nucleotide sequence at the genomic DNA level.

A total of eight SNPs were found to be significantly associated with seed yield in tepary bean in the genome-wide association study. However, these SNPs had low R-square values (<5%), indicating that tepary bean yield can be controlled by multiple genes with minor effects. Similar findings were also reported in other crops such as soybean [27] and cowpea [30]. One annotated gene, *Phacu.CVR.009G294200.1,* was found 20 kb upstream of Ph_Chr09_37443441. This annotated gene encodes for a peroxidase superfamily protein. Previous studies have reported a role of this gene in increasing seed yield. For example, it has been demonstrated that the overexpression of this gene resulted in an increase of seed yield of up to 50% in soybean [31]. Therefore, the results suggest that *Phacu.CVR.009G294200.1* can control seed yield in tepary bean. The eight SNPs associated with seed yield can be implemented in a tepary bean program to select for breeding lines with a higher yield potential. Therefore, preliminary yield selection can be conducted in mid-stage of the tepary bean breeding pipeline. However, these SNPs should be validated prior to their implementation and use in population development and selection of elite tepary bean lines.

## 4. Materials and Methods

### 4.1. Plant Materials and Phenotyping

A total of 153 tepary bean accessions obtained from the United States Department of Agriculture (USDA), Pullman, Washington, were evaluated for 100-seed weight and seed yield in Chillicothe, Texas, in 2021 and 2022. The experiment was organized in a randomized complete block design with four blocks. The experimental unit was defined by two rows that were 5 m long, with 0.7 m spacing between rows. Plant spacing within each row was about 13 cm. Herbicides were applied prior to planting to control weeds. Middle crop sweeps were run to control weeds during the growing season. Irrigation was applied at a rate of 51 mm every month when rainfall was not sufficient. For the 2021 season, the planting and harvesting dates were May 13 and August 28, respectively. For the 2022 season, the planting and harvesting dates were June 7 and September 20, respectively. Yield was obtained by harvesting the two-row plots from each experimental plot. Hundred-seed weight was obtained using three samples of 100 seeds from each experimental plot, and the average weight between the 3 samples was used for the analysis. Phenotypic data were analyzed using analysis of variance (ANOVA) in JMP Genomics^®^ 7 (SAS Institute, Inc, Cary, NC, USA). Mean separation analysis was conducted using a Fisher’s protected LSD at α = 0.05. The Least Square Means (LS Means) of the combined 2021 and 2022 data were used for genome-wide association study (GWAS).

### 4.2. Genotyping

DNA was extracted from young and fresh tepary bean leaves. DNA was extracted from leaf tissues using the CTAB (hexadecyltrimethyl ammonium bromide) procedure [32]. The extracted DNA was quantified using a NanoDrop 200c spectrophotometer (Thermo SCIENTIFIC, Wilmington, DE, USA). High-quality DNA samples were sent to the Texas A&M Genomics & Bioinformatics service (https://www.txgen.tamu.edu/, accessed on 20 May 2024) for whole-genome resequencing. DNA sequencing was conducted using a NovaSeq 6000 S4 X series (Illumina ®, San Diego, CA, USA). Raw sequences were processed to discard adaptor contaminants and low-quality reads. The reads were aligned to the tepary genome [14]. Bam files were established and used as inputs for downstream analyses. SNP assembly, mapping, and discovery were conducted using the Genome Analysis Toolkit (GATK) pipeline (https://gatk.broadinstitute.org/hc/en-us, accessed on 20 May 2024). Initial SNP calls were also conducted using GATK. Tepary bean genotypes with more than 10% missing data were removed. SNP calls with a heterozygous rate > 10% and minor allele frequency < 5% were also removed from the analysis. After SNP filtering, a total of 230,000 high-quality SNPs were used for the genome-wide association study.

### 4.3. Population Structure and Genetic Diversity

The tepary bean population structure (K) was inferred using an admixture model in STRUCTURE 2.3.4 [33]. To estimate K, ten runs were analyzed independently. The burn-in period Markov Chain Monte Carlo (MCMC) length was set to 20,000, and the number of MCMC iterations was 20,000 during the burn-in phase. Delta K and optimum K were estimated using STRUCTURE Harvester [34]. A Q matrix consisting of K vectors was established. Each tepary bean accession was assigned to a Q group using a 0.55 probability threshold [12]. A structure graph was established using the ‘Sort by Q’ option and the ‘Structure plot’ function in STRUCTURE 2.3.4 [33]. Genetic diversity analysis was conducted using a maximum likelihood tree model and run in MEGA 7 [35]. The Q matrix was combined with the genetic diversity tree. In MEGA 7, each Q group color was similar to the Q plots obtained from the population structure using the ‘branch line’ and ‘node/subtree marker’ functions [12].

### 4.4. Genome-Wide Association Study (GWAS) and Candidate Gene Discovery

**Figure 6:**
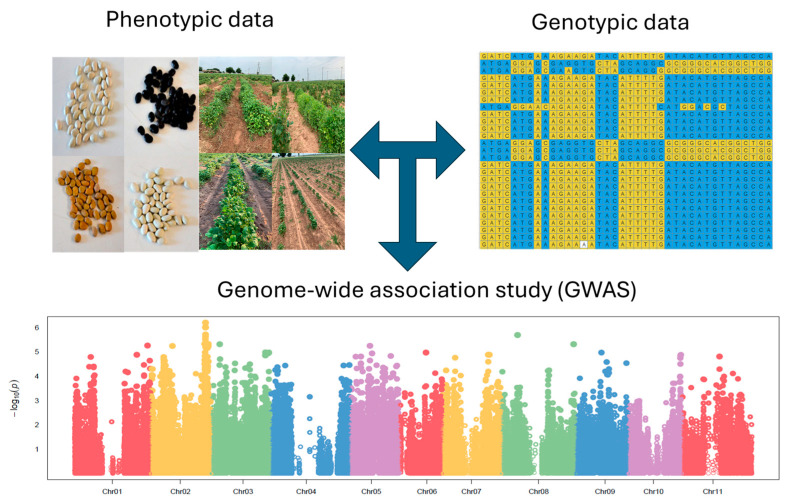
Schematic overview of the approach for genome-wide association study for 100-seed weight and seed yield in tepary bean.

The genome-wide association study was conducted using a mixed linear model and a compressed linear model using the “Genome Association and Prediction Integrated Tool” (GAPIT) function [36] in R. Both models account for the population structure to reduce false positive discoveries [36]. A total of 230,000 high-quality SNPs from the whole-genome resequencing were used to conduct the GWAS. The LOD [−log_10_(*p*-value)] thresholds for 100-seed weight and seed yield were 5.00 and 3.80, respectively [12]. Candidate gene discovery was conducted by scanning a 50 kb region of the most significant SNP between the two models [12]. The candidate gene search was performed using Phytozome v 13 (https://phytozome-next.jgi.doe.gov/, accessed on 20 May 2024). The overall approach used for this study is shown in Figure 6.

## 5. Conclusions

This study collected data on 100-seed weight and seed yield of tepary bean. The phenotypic data were from a two-year field experiment in Chillicothe, TX, USA. The results indicated a large variation in these traits, suggesting that the population being used for this study is suitable for a genome-wide association study for 100-seed weight and seed yield. Parents with a larger seed size and higher seed yield were identified in this study. These parents can be used to improve tepary bean or to build interspecific lines between tepary bean and common bean. SNP markers associated with 100-seed weight and seed yield were also identified. A total of 5 and 20 SNPs were found using a mixed linear model (MLM) and compressed mixed linear model (cMLM), respectively, for 100-seed weight. A total of one and eight SNPs were identified to be associated with seed yield using an MLM and cMLM, respectively. These SNP markers can be used for molecular breeding of tepary bean. Candidate genes with functional annotations relevant to seed development and seed yield were also identified.

## Figures and Tables

**Table 1:** Significant single-nucleotide polymorphism (SNP) markers associated with 100-seed weight in tepary bean using mixed linear model (MLM) and compressed MLM.

Model	SNP	Chromosome	Position	LOD log_10_(*p*-Value)]	Minor Allele Frequency	R_Square (%)
MLM	Ph_Chr02_41007928	Chr02	41007928	5.04	0.45	12.72
	Ph_Chr02_41008221	Chr02	41008221	5.32	0.45	13.66
	Ph_Chr02_41008236	Chr02	41008236	5.32	0.45	13.66
	Ph_Chr02_41016239	Chr02	41016239	5.50	0.45	14.16
	Ph_Chr03_29040578	Chr03	29040578	5.13	0.48	17.69
cMLM	Ph_Chr01_54553950	Chr01	54553950	5.26	0.45	16.66
	Ph_Chr02_16248843	Chr02	16248843	5.25	0.45	15.28
	Ph_Chr02_40340436	Chr02	40340436	5.33	0.46	11.69
	Ph_Chr02_40901629	Chr02	40901629	5.52	0.45	12.97
	Ph_Chr02_41007928	Chr02	41007928	5.72	0.45	13.28
	Ph_Chr02_41008221	Chr02	41008221	6.01	0.45	14.22
	Ph_Chr02_41008236	Chr02	41008236	6.01	0.45	14.22
	Ph_Chr02_41008249	Chr02	41008249	5.20	0.46	12.25
	Ph_Chr02_41008429	Chr02	41008429	5.63	0.45	13.22
	Ph_Chr02_41016239	Chr02	41016239	6.20	0.45	14.66
	Ph_Chr02_42360227	Chr02	42360227	5.19	0.47	11.78
	Ph_Chr02_42377727	Chr02	42377727	5.16	0.45	11.88
	Ph_Chr02_42378284	Chr02	42378284	5.16	0.45	11.88
	Ph_Chr02_43137572	Chr02	43137572	5.23	0.46	10.66
	Ph_Chr02_43159031	Chr02	43159031	5.33	0.46	12.13
	Ph_Chr03_6452621	Chr03	6452621	5.32	0.45	15.88
	Ph_Chr05_15111527	Chr05	15111527	5.25	0.45	14.78
	Ph_Chr08_11898325	Chr08	11898325	5.70	0.45	17.19
	Ph_Chr08_53883571	Chr08	53883571	5.32	0.45	15.88
	Ph_Chr08_53883585	Chr08	53883585	5.32	0.45	15.88

**Table 2:** Significant single-nucleotide polymorphism (SNP) markers associated with seed yield in tepary bean using mixed linear model (MLM) and compressed MLM.

Model	SNP	Chromosome	Position	LOD [−log_10_(*p*-Value)]	Minor Allele Frequency	R_Square (%)
MLM	Ph_Chr09_37443441	Chr09	37443441	3.81	0.47	7.33
cMLM	Ph_Chr09_37317875	Chr09	37317875	3.87	0.46	6.10
	Ph_Chr09_37317968	Chr09	37317968	3.87	0.46	6.10
	Ph_Chr09_37321813	Chr09	37321813	3.94	0.46	6.15
	Ph_Chr09_37324571	Chr09	37324571	3.86	0.45	6.08
	Ph_Chr09_37332586	Chr09	37332586	3.89	0.5	6.10
	Ph_Chr09_37332627	Chr09	37332627	3.82	0.49	6.02
	Ph_Chr09_37443441	Chr09	37443441	3.90	0.47	6.12
	Ph_Chr09_37443547	Chr09	37443547	4.01	0.45	6.21

## Data Availability

Data are contained within the article.
